# Mortality and Disease Burden of Injuries from 2008 to 2017 in Anhui Province, China

**DOI:** 10.1155/2020/7303897

**Published:** 2020-04-21

**Authors:** Xiu-Ya Xing, Peng Wang, Zhiwei Xu, Qin He, Rui Li, Ye-Ji Chen, Li-Na Liu, Yan-Mei Mao, Chan-Na Zhao, Yi-Lin Dan, Qian Wu, Hai-Feng Pan, Zhi-Rong Liu, Wenbiao Hu

**Affiliations:** ^1^Department of Chronic Non-communicable Disease Prevention and Control, Anhui Provincial Center for Disease Control and Prevention, Hefei, Anhui 230601, China; ^2^Center for Genetic Epidemiology and Genomics, School of Public Health, Medical College of Soochow University, 199 Renai Road, Suzhou, Jiangsu, China; ^3^School of Public Health and Social Work, Institute of Health and Biomedical Innovation, Queensland University of Technology, Brisbane, Queensland, Australia; ^4^Department of Epidemiology and Biostatistics, School of Public Health, Anhui Medical University, 81 Meishan Road, Hefei, Anhui 230032, China

## Abstract

**Objective:**

To investigate the temporal trends in mortality and disease burden of injuries in Anhui province from 2008 to 2017, so as to provide reference for injury control and prevention.

**Methods:**

Data of mortality were collected from 9 national surveillance points in Anhui province during 2008-2017 in the Information System for Death Cause Register and Management. The surveillance data were analyzed by using crude mortality, standardized mortality rate (SMR), potential year of life lost (PYLL), PYLL rate (PYLLR), and average of year life lost (AYLL).

**Results:**

There were a total of 44855 people died from injury, accounted for 9.44% of the all-cause mortality, ranked as the fifth leading cause of deaths in the whole population, and denoted the first leading cause of deaths in the 0-44 year's group. The leading causes of injury deaths were road traffic accidents, suicide, accidental falls, drowning, and poisoning. Road traffic accidents was the primary cause of injury deaths among the male population, while suicide was the dominate cause of injury deaths among the female population. Drowning, traffic accidents, and suicide accounted for the most injury deaths among the population aged 0-14 years, 15-64 years, and above 60 years, respectively. The road traffic accidents accounted for the largest proportion of injury PYLL and PYLLR, and drowning caused the highest AYLL among injury deaths.

**Conclusion:**

In Anhui province, road traffic accidents, suicide, accidental falls, drowning, and poisoning were the top five causes of injury deaths that harm the health of local residents; corresponding injury prevention strategies should be formulated.

## 1. Introduction

Injury, communicable diseases, and chronic non-communicable diseases are listed as the three major burdens of disease worldwide [1]. Road traffic accidents, suicide, accidental falls, drowning, and accidental poisoning are the common types of injury [2–4]. It has been estimated that there are more than 5 million people died from injuries each year worldwide [5, 6]. Although the majority of deaths caused by injuries (over 90%) occurred in low- and middle-income countries, various types of injuries collectively have become an increasing public health issue that threaten the health of people and rank as the fifth cause of deaths in China [2, 7–9]. From 1995 to 2008, it has been reported that there were about 0.70 to 0.75 million people who died from injuries each year, accounted for 9% of all-cause mortality in China [10, 11]. In addition to the serious harms of injury on people's life and health, the consequently heavy financial burden cannot be ignored [12], as the total economic burden caused by injury is more than 379.231 billion Yuan in China [13].

The network report system that recorded the mortality data has been established in China since 2008. Anhui, a province located in the eastern region of China, is the 22^nd^ largest Chinese province based on area, the 8^th^ most populous province, and the 12^th^ most densely populated region in the whole country. In Anhui province, the mortality statistics is often generated from cross-sectional population based surveys, and the related trend analysis has not been carried out yet.

In the present study, in order to derive a precise evaluation on the status regarding injury mortality and provide reference for formulating injury control and prevention strategies, we investigated the temporal trends in mortality and disease burden of injury in Anhui province from 2008 to 2017. In addition, we further performed a detailed analysis of the leading causes of injury-related mortality.

## 2. Methods

### 2.1. Data Sources

In the present study, data of mortality were obtained from the Information System for Death Cause Register and Management covering 9 national surveillance points in Anhui province from year of 2008 to 2017. The nine national surveillance points included Juchao district of Hefei, Yushan district of Maanshan, Tianchang city of Chuzhou, Jing county of Xuancheng, Mengcheng county of Bozhou, Shouxian county of Luan, Lingbi county of Suzhou, Yingdong district of Fuyang, and Yongqiao district of Suzhou, where the location of mentioned the 9 surveillance points was evenly distributed within Anhui province. The nine surveillance points have a good quality control, the mortality rate is above 5.5‰, and the correct encoding rate is 98.00%. The population demographic data were retrieved from the Resident Population Information System of Chinese Center for Disease Control and Prevention (China CDC).

This study was approved by the ethical committee of Anhui Provincial Center for Disease Control and Prevention. Data obtained from the reporting system did not have any personal identifiers; privacy and confidentiality of all information were maintained.

### 2.2. Classification of Injury Death

The classification of injury death was based on the criteria of the International Statistical Classification of Diseases and Related Health Problems (WHO), Tenth Revision (ICD-10), edition 2 [14, 15]. Damage (V01-Y89) includes accidental injury (V01-X59, Y40-Y86, Y88, and Y89) and intentional injury (X60-Y09, Y35-Y36, Y87.0, and Y87.1); accidental injury represents road traffic accidents (V01-V04, V06, V09-V80, V87, V89, and V99), accidental poisoning (X40-X49), accidental falls (W00-W19), fire disaster (X00-X09), drowning (W65-W74), and other accidental injuries (V05, V07-V08, V81-V86, V88, V90-V98, W20-W64, W75-W99, X10-X39, X50-X59, Y40-Y86, Y88, and Y89); intentional injury includes suicide and its sequelae (X60-X84, Y87.0), homicide and its sequelae (X8-Y09, Y87.1), war (Y36), and other intentional injuries (Y35).

### 2.3. Statistical Analysis

Data arrangement and analysis were implemented by Microsoft Excel and SPSS 23.0 statistical software. The epidemiological characteristics of injury death and trends were analyzed and assessed by crude mortality rate, standardized mortality rate (SMR), composition ratio, rank order of cause of death, and the annual percentage change in mortality rate (APC). The comparison of mortality rate between two groups was performed using *Z* test, with *p* < 0.05 for statistical significance.

To emphasize the influence of injury caused early death on the life reducing, the following terms were applied to evaluate the disease burden of injury, including potential years of life lost (PYLL), PYLL rate (PYLLR), and average years of life lost (AYLL). The SMR was calculated according to the 6^th^ national population census of China. According to the maximum expected value method, the life expectancy of 70 years was regarded as the assumed life expectancy to calculate the relevant indicators of life reduction. The age range of “premature death” was from 1 to 69 years old. The calculation method was as follows:

(1) $$\begin{array}{c} \text{Mortality rate}=\text{the number of deaths}/\text{total population}\times 100000/100\text{ thousand},\\ \text{APC}=\left(10^{\text{a}}-1\right)\times 100\%, \end{array}$$

where the logarithm of mortality in each year is the dependent variable and year is the independent variable; the fitting line was *Y* = *b* + *ax*, the value of *a* was obtained, *Y* = log (mortality rate), and *x* = year. Then the hypothesis test is carried out on APC to check whether APC is an accidental change.

PYLL = ∑ (life expectancy - median for each age group - 0.5) × number of deaths in each age group.

(2) $$\begin{array}{c} \text{PYLLR}=\text{PYLL}/n\times 1,000\text{‰}\left(n\text{ is the total population age from }1\text{ to }69\text{ years in the target population}\right),\\ \text{AYPLL}=\text{YPLL}/d\left(d\text{ is the total number of deaths from a cause of death in the target population aged from }1\text{ to }69\text{ years}\right). \end{array}$$

## 3. Results

### 3.1. Injury Deaths Profile

From 2008 to 2017, a total of 74,225,139 people (37,509,558 males and 36,715,581 females) were recruited within 9 national surveillance points in Anhui province, and 44855 people (30213 males and 14642 females) died from injuries, with an average crude mortality rate of 60.43/100000 (SMR: 59.50/100000), accounting for 9.44% of the all-cause mortality (44855/475177). In terms of gender distribution of injury mortality, the average male crude injury mortality rate was 80.55/100000 (SMR: 82.53/100000), accounting for 10.90% of the all-cause mortality in males (30213/277073), and the average female crude injury mortality rate was 39.88/100000 (SMR: 36.98/100000), accounting for 7.39% of the all-cause mortality in females (14642/198104). The difference of injury SMR between the male and female groups was statistically significant (*Z* = 80.13, *p* < 0.001), with an increased injury SMR in male than in female groups. The injury death ranked 5^th^ of causes of death after cerebrovascular disease, malignant tumor, and heart and respiratory diseases, and denoted the first cause of death in the group of age 0-44 years.

### 3.2. Cause of Injury Deaths

In Anhui province, from 2008 to 2017, the top five causes of injury deaths were road traffic accidents, suicide, accidental falls, drowning, and accidental poisoning, accounting for 87.26% cause of injury deaths. The first leading cause of injury deaths for males was road traffic accidents, followed by suicide; while for females, suicide was the leading mechanism of injury deaths, followed by road traffic accidents; the other rank order of cause of injury deaths in both males and females was consistent (Table 1).

The rank order of cause of injury deaths in different age groups showed significant differences, among which road traffic accidents, drowning, and suicide were the top three causes of injury deaths among each age subgroup. Drowning was the leading cause of injury deaths in the age group of 0-14 years old and contributed for 41.78% of the cause of injury deaths; moreover, road traffic accidents were the first leading cause of injury deaths in the age group of 15-44 and 45-64 years old (49.07% and 43.29%). Suicide was the first leading cause of injury deaths in the age group above 65 years old and accounted for 36.62% of the cause of injury deaths in this age group. In addition, road traffic accidents ranked as the second cause of injury deaths in the age group of 0-14 and over 65 years old (25.54% and 23.57%). Furthermore, suicide was the second rank cause of injury deaths in the age group of 15-44 and 45-64 years old (17.12% and 23.47%). Accidental fall-caused death in the age group 65 and older accounted for 52.93% of the total number of accidental fall death in all age groups (Table 1).

### 3.3. Trend of Injury Mortality Rates in Different Genders

During the past 10 years (2008-2017), the injury mortality rate of residents in Anhui province declined by 4.72% each year. The decrease of SMR between male and female groups was statistically significant (*p* < 0.001), with the SMR of 4.28% in males and 5.81% in females. When the SMR is compared between different genders in the same year, there was a higher SMR in male group than those of female group (all *p* < 0.05) (Table 2).

### 3.4. Trends in Injury Mortality Rates in Different Age Groups

**Figure 1:**
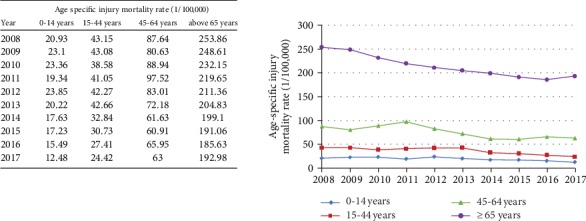
The trends of age-specific injury mortality rates (1/100000) in Anhui province from 2008 to 2017.

**Figure 2:**
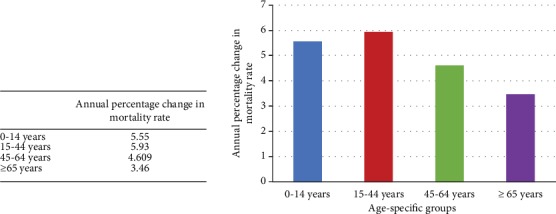
Annual percentage changes in mortality rate among different age groups from 2008 to 2017.

From 2008 to 2017, the trends of age-specific injury mortality rate showed a steady decline (Figure 1). The age group of 15 to 44 years old had the greatest decreasing rate of APC, and the age group of above 65 years old had the lowest decreasing APC, as compared with other age groups (Figure 2).

### 3.5. Trends in Major Injury Mortality Rates

From 2008 to 2017, the trend of the top five injury mortality rates was different, among which the SMR of road traffic accidents, suicides, and accidental falls displayed a significant downward trend (all *p* < 0.05), with the APC of 4.19%, 8.16%, and 2.66%, respectively. Furthermore, the road traffic accidents SMR peaked in 2011 and then showed a downward trend, with the largest decline in 2013 to 2014. However, the trend of SMR in drowning and accidental poisoning was not significant (Table 3).

### 3.6. The Evaluation of Disease Burden in Injury Deaths

During the past 10 years, the PYLL was 830147 person-years attributed to injury and accounted for 33.89% of all-cause mortality (2449302 person-years); the PYLLR was 12.15% and AYLL was 26.33 years, and the results indicated that injury ranked 1^st^ of all-cause mortality. When analyzing the gender-specific disease burden, the PYLLR was higher in male group than in female group (17.41% *vs.* 6.74%); although the difference regarding AYLL was only 0.70 years between male and female groups, the injury PYLL in male was 2.65 times higher than in female (602722 person-years *vs.* 227425 person-years). The top five causes of injury deaths contributed in PYLL were road traffic accidents, suicides, drowning, accidental falls, and accidental poisoning. While, the top five causes of injury deaths in AYLL were drowning, homicide, road traffic accidents, fire disaster, and accidental poisoning (Table 4).

## 4. Discussion

In comparison to the frequency on occurrence of injury, injury deaths are only the “tip of the iceberg.” It has been estimated that the injury deaths and occurrence ratio is about 1 : 100 in China. Injury is characterized as high incidence rate, high disability rate, and high mortality rate, as well as an often-neglected common health issue.

In our study, we revealed that, from 2008 to 2017, the average crude injury mortality rate of residents was 60.43/100000 (SMR: 59.50/100000) in Anhui province and ranked 5^th^ of all-cause mortality. As compared with other provinces, there was an increased SMR in Anhui province (59.50/100000) than in Chongqing (55.57/100000), Guangxi Zhuang autonomous region (46.45/100000), Guangdong province (43.11/100000), Wuxi city (38.62/100000), and nationwide (44.98/100000) [16–20]; however, the SMR of Anhui province was similar to that in Taizhou (60.65/100000) [21]. In Anhui province, injury mortality rates displayed a descending trend year by year from 2008 to 2017, and this trend was consistent with the national and some regional reports. The top three leading causes of injury deaths were road traffic accidents, suicide, and accidental falls; this mortality spectrum of injuries in Anhui province was also similar to the national situation.

The SMR of injury mortality were higher in male than in female groups each year, as well as a 2.65 times increase of PYLL and 2.58 times PYLLR in male than in female groups; this result was consistent with previous studies [20–23], suggesting the higher injury burden in male. In fact, PYLL, PYLLR, and other indicators focused on the description of premature death, emphasizing the loss of life caused by premature death [22, 24]. The increased SMR of injury deaths, PYLL, and PYLLR in male may be related to the fact that they have to take much more social responsibilities and usually work on physical labor that made them in higher risk-taking behaviors than female [17, 25, 26].

Road traffic accidents were the first cause of injury deaths and the most important cause of life loss in Anhui province. The PYLL of road traffic accidents was 368017.5 years, accounting for 44.33% of all PYLL caused by injury. It may be explained by a rapid increase in the number of vehicles over the past few years, with inevitable surges in accidents and the traffic jam, as well as the lower safety awareness [27, 28]. Furthermore, the SMR of road traffic accidents present two turning points: one is the downtrend year by year after the peak in 2011 and the other is that there was the largest annual descend range from 2013 to 2014. One important underlying reason for this change of trends may be explained by the promulgation of “China's newly-amended Road Traffic Safety Law” which took effect on May 2011. The newly implemented amendment law relatively imposed new stiff penalties, especially criminal charges on persons with a drunk driving. In all, the improvement of public awareness on traffic safety and road conditions and promulgation of relevant laws and regulations are effective measures to reduce traffic fatal-accident rate.

Suicide was the second cause of injury deaths in Anhui province and the first cause of injury death in women, with a higher AYLL of suicide in women than in men; this is closely associated with the special psychological and physiological characteristics of women. Studies have revealed that, as compared with men, women have a higher neuroticism score and are inclined to take negative coping strategies; thus, they are easily to have a suicidal tendency [18, 29]. Moreover, we also found that suicide ranked 1^st^ of injury deaths in age above 65 years old, which was similar to the results of Shandong province, Guangxi province, and Hunan province in China [22, 30]. Several studies have demonstrated that suicide has a relationship with psychological status, family conflicts, and healthy condition. The elderly people, as a vulnerable group, gained fewer social supports and were more likely to have a suicidal behavior because of family conflicts and diseases. In recent years, the suicide rate in Chinese rural elderly people has been increasing [31]; therefore, the government health care/service and family psychological health education should be allocated to improve the psychological status on both female and elder people, to reduce the incidence/mortality rate of suicide.

Accidental falls were the third cause of injury deaths in all age groups, among which the age group above 65 years old showed a higher incidence of accidental fall deaths than the other age groups, accounting for 17.48% of accidental fall deaths. The study performed by Shao et al. also suggested that elder people are prone to accidental falls [32]. Anhui is one of the main sources of rural labor service export. The phenomenon of left-behind elderly people in rural areas has become a common social issue, such that many elderly people in rural areas have to do housework, do farm work, and help their children babysit the new born, but the lack of family care on elderly people makes them easily fall down. Therefore, more attention and care should be paid for the elder people to prevent the possible accidental falls and improve their quality of life.

Drowning represented the leading cause of injury deaths in children age group of 0-14 years and is the first rank cause of injury deaths in the whole population with premature death. In recent years, incidence of children drowning has been reported frequently [33, 34]. The risk factors that contributed to the child drowning included children personality characteristics, family factors (inadequate parental supervision and control), and environmental factors (shortness of life-saving equipment and safe guards) [33, 35]. Children drowning cognitive education should be strengthened to enhance their safety awareness; first-aid knowledge and skills for drowning should be provided to improve children's capability in facing emergent situations [36].

There are some limitations that have to be taken into account. First, the incomplete data of Information System for Death Cause Register and Management may underestimate the true injury mortality. Second is the lack of information on potential influencing factors, such as socio-economic status, family background, or detailed conditions; thus, the association of injury deaths with potential influencing factors could not be fully understood.

Injury is one of the main causes of deaths in the residents of Anhui province with age and gender heterogeneity. Traffic accidents, suicide, falls, drowning, and poisoning were the top five causes of deaths and should be targeted for injury prevention activity. Moreover, it is critically pivotal to improve policies and programs that can deliver effective measures in the high-risk populations and areas.

## Figures and Tables

**Table 1:** The proportion and ranks of cause of injury death in Anhui province from 2008 to 2017.

Injury types	Male		Female		0~14 years		15~44 years		45~64 years		>65 years		Overall	
	Rank	Deaths (proportion %)	Rank	Deaths (proportion %)	Rank	Deaths (proportion %)	Rank	Deaths (proportion %)	Rank	Deaths (proportion %)	Rank	Deaths (proportion %)	Rank	Deaths (proportion %)
Road traffic accidents	1	12336 (40.86)	2	4224 (28.85)	2	678 (25.54)	1	6443 (49.07)	1	5654 (43.29)	2	3785 (23.57)	1	16560 (36.94)
Accidental poisoning	5	1378 (4.53)	5	472 (3.23)	4	90 (3.34)	5	576 (4.41)	4	703 (5.38)	5	481 (2.94)	5	1850 (4.11)
Accidental falls	3	3399 (11.23)	3	1883 (12.8)	3	106 (3.98)	3	990 (7.50)	3	1390 (10.61)	3	2796 (17.48)	3	5282 (11.74)
Fire disaster	6	389 (1.29)	6	210 (1.44)	6	27 (1.02)	7	54 (0.41)	6	91 (0.70)	6	427 (2.68)	6	599 (1.34)
Drowning	4	2601 (8.56)	4	1659 (11.31)	1	1114 (41.78)	4	925 (6.97)	5	701 (5.31)	4	1520 (9.51)	4	4260 (9.46)
Suicide	2	6229 (20.65)	1	4961 (33.94)	5	50 (1.90)	2	2242 (17.12)	2	3062 (23.47)	1	5836 (36.62)	2	11190 (24.99)
Homicide	7	238 (0.79)	7	120 (0.81)	6	27 (1.02)	6	222 (1.69)	7	79 (0.60)	7	30 (0.19)	7	358 (0.80)
Other intentional injuries		3643 (12.09)		1113 (7.62)		568 (21.4)		1687 (12.84)		1387 (10.62)		1114 (7.01)		4756 (10.63)
Overall		30213 (100.00)		14642 (100.00)		2660 (100.00)		13139 (100.00)		13067 (100.00)		15989 (100.00)		44855 (100.00)

**Table 2:** The trends of gender-specific injury mortality rates (1/100000) in Anhui province from 2008 to 2017.

Year	Male			Female			Overall		
	Deaths	Crude mortality rate	SMR	Deaths	Crude mortality rate	SMR	Deaths	Crude mortality rate	SMR
2008	3286	83.65	97.82^a^	1659	44.69	48.32	4945	64.72	72.91
2009	3230	81.81	94.94^a^	1672	44.84	47.92	4902	63.85	70.86
2010	3268	82.51	93.43^a^	1531	40.86	43.16	4799	62.26	68.22
2011	3251	89.22	98.19^a^	1578	44.30	43.34	4829	67.01	69.80
2012	3289	90.88	90.32^a^	1550	43.00	38.38	4839	66.99	63.90
2013	3138	86.72	88.43^a^	1443	40.04	37.19	4581	63.42	62.52
2014	2729	74.53	75.15^a^	1281	35.47	32.25	4010	55.13	53.52
2015	2681	74.13	73.64^a^	1273	34.27	31.04	3954	53.93	51.84
2016	2740	73.66	71.10^a^	1352	36.27	30.69	4092	54.94	50.62
2017	2601	68.58	67.83^a^	1303	35.19	30.86	3904	52.08	49.17
Overall	30213	80.55	82.53	14642	39.88	36.98	44855	60.43	59.50
APC (%)			-4.28			-5.81			-4.72
*t*			-7.71			-9.42			-10.68
*p*			<0.001			<0.001			<0.001

^a^Compared with the female in the same year (*p* < 0.05). APC: annual percentage change in mortality rate; SMR: standardized mortality rate.

**Table 3:** The top 5 injury mortality rates (1/100000) and its variation trend in Anhui province from 2008 to 2017.

Year	Road traffic accidents			Suicide			Accidental falls			Drowning			Accidental poisoning		
	Deaths	Crude mortality rate	SMR	Deaths	Crude mortality rate	SMR	Deaths	Crude mortality rate	SMR	Deaths	Crude mortality rate	SMR	Deaths	Crude mortality rate	SMR
2008	1741	22.79	24.71	1365	17.87	20.59	513	6.71	8.78	433	5.67	6.11	197	2.58	2.84
2009	1843	24.01	25.70	1393	18.15	20.64	414	5.39	7.03	411	5.35	5.56	193	2.51	2.76
2010	1832	23.77	25.13	1255	16.28	18.43	476	6.18	7.60	432	5.60	5.79	195	2.53	2.73
2011	1937	26.88	27.95	1197	16.61	17.01	529	7.34	8.12	405	5.62	5.67	173	2.40	2.52
2012	1863	25.79	25.32	1121	15.52	14.42	595	8.24	7.41	454	6.28	5.81	182	2.52	2.54
2013	1692	23.43	24.02	1115	15.44	14.65	564	7.81	7.42	426	5.90	5.74	180	2.49	2.47
2014	1457	20.03	20.57	1016	13.97	13.00	493	6.78	6.27	388	5.33	5.16	159	2.19	2.09
2015	1336	18.22	18.36	1019	13.90	12.83	517	7.05	6.26	483	6.59	6.30	175	2.39	2.36
2016	1403	18.84	18.40	926	12.43	10.90	584	7.84	6.49	414	5.56	5.04	224	3.01	2.98
2017	1456	19.42	19.40	783	10.45	9.48	597	7.96	6.92	414	5.52	5.12	172	2.29	2.24
Overall	16560	22.31	22.57	11190	15.08	14.58	5282	7.12	6.84	4260	5.74	5.48	1850	2.49	2.50
APC (%)			-4.19			-8.16			-2.66			-1.29			-1.75
*t*			-4.35			-16.04			-3.09			-1.76			-1.57
*p*			0.002			<0.001			0.015			0.116			0.156

APC: annual percentage change in mortality rate; SMR: standardized mortality rate.

**Table 4:** Comparisons of PYLL, PYLLR, and AYLL in different types of injury deaths in Anhui province from 2008 to 2017.

Injury types	Male			Female			Overall		
	PYLL (person-years)	PYLLR (‰)	AYLL (years)	PYLL (person-years)	PYLLR (‰)	AYLL (years)	PYLL (person-years)	PYLLR (‰)	AYLL (years)
Road traffic accidents	279054	8.06	26.36	88963.5	2.64	26.14	368017.5	5.38	26.31
Accidental poisoning	27909.5	0.81	24.12	8927	0.26	27.64	36836.5	0.54	24.89
Accidental falls	52755.5	1.52	23.18	10484	0.31	22.74	63239.5	0.93	23.11
Fire disaster	3662.5	0.11	25.98	1413.5	0.04	24.37	5076	0.07	25.51
Drowning	82882.5	2.39	41.67	34482.5	1.02	36.15	117365	1.72	39.88
Suicide	67092.5	1.94	19.67	3795.5	0.11	35.47	130542.5	1.91	20.57
Homicide	7758	0.22	33.44	3715.5	0.11	35.39	11553.5	0.17	34.08
Other intentional injuries	81607.5	2.36	27.83	15909	0.47	28.16	97516.5	1.43	27.89
Overall	602722	17.41	26.52	227425	6.74	25.82	830147	12.15	26.33

PYLL: potential year of life lost; PYLLR: PYLL rate; AYLL: average of year life lost.

## Data Availability

No additional data are available.
